# The Haering Patent

**Published:** 1881-09

**Authors:** 


### THE HAERING PATENT.

"Is there danger of the Goodyear Company reviving the Haering patent so as to make an extension of their time of license?"

No: If the Goodyear Company had not in 1868 proved, in the United States Circuit Court of R. I., that this patent was an infringement on theirs, so that the court declared it null and void, they might use it now for five years to come. But though they subsequently bought the Haering patent they cannot use it.—*Items of Interest.*

